# Effects of oxymatrine on the proliferation of human liver cancer Bel-7404 cells: A protocol of systematic review and meta-analysis

**DOI:** 10.1097/MD.0000000000020181

**Published:** 2020-06-05

**Authors:** Jing Li, Zhi-Ye Liu, Hai-Bo Yu, Qing Xue, Xiu-Sheng Qu

**Affiliations:** a Department of Physiology, Jiamusi University School of Basic Medical Sciences; b Department of Chemotherapy and Radiotherapy, First Affiliated Hospital of Jiamusi University; c Department of Cardiology, First Affiliated Hospital of Jiamusi University; d Clinical Medicine of Class 7 in Grade 2016, Jiamusi University, Jiamusi, China.

**Keywords:** effect, human liver cancer Bel-7404 cells, oxymatrine

## Abstract

**Background:**

This study will examine the effects of oxymatrine on the proliferation of human liver cancer Bel-7404 cells (HLCBC).

**Methods:**

This study will search electronic bibliographic databases available in PUBMED, EMBASE, Cochrane Library, Scopus, Cumulative Index to Nursing and Allied Health Literature, China Biology Medicine, and China National Knowledge Infrastructure. We attempt to search case-controlled studies (CCSs) or randomized controlled studies (RCSs) pertaining to HLCBC from their inception to the February 29, 2020 without limitations of language and publication time. We will include any CCSs or RCSs on exploring oxymatrine on the proliferation of HLCBC. We will assess the methodological quality of CCSs by Newcastle-Ottawa Scale, and RCSs by Cochrane risk of bias tool. Review Manager 5.3 software will be utilized for statistical analysis.

**Results:**

The current study will summarize most recent eligible studies to investigate the effects of oxymatrine on the proliferation of HLCBC.

**Conclusion:**

Its results may provide reliable scientific evidence on effects of oxymatrine on the proliferation of HLCBC.

**Systematic review registration:**

INPLASY202040026.

## 1 Introduction

Liver cancer (LC) is one of the most major health problems,^[1,2]^ which is the 4th leading cause of cancer mortality around the world.^[3–6]^ Although the diagnosis methods of LC have advanced and its therapy has been improved significantly in the past decades, the curative effects of existing chemotherapeutic drugs are still not satisfied.^[7–13]^ Thus, it is still very important to search highly efficient antitumor drugs.

Apoptosis plays a very vital role in various processes, including embryonic development, and chemical-induced cell death. It also represents a physiological way to get rid of excess cells during LC development and regeneration.^[14,15]^ Previous studies indicated that oxymatrine had an effect on the proliferation of human liver cancer Bel-7404 cells (HLCBC).^[16–19]^ However, there are inconsistent results among those studies. Thus, this systematic review will examine the effects of oxymatrine on the proliferation of HLCBC.

## 2 Methods

### 2.1 Study registration

We have registered this study on INPLASY202040026, and have organized it based on the Preferred Reporting Items for Systematic Reviews and Meta-Analysis (PRISRMA) Protocol statement guidelines.^[20,21]^

### 2.2 Eligibility criteria

#### 2.2.1 Types of trials

This study will include case-controlled studies (CCSs) or randomized controlled studies (RCSs) that assessed the effects of oxymatrine on the proliferation of HLCBC.

#### 2.2.2 Types of subjects

This study will include HLCBC as its research target.

#### 2.2.3 Types of interventions

All studies utilized oxymatrine to manage HLCBC in the experimental group.

All studies which used any treatments as their comparators will be included, except oxymatrine.

#### 2.2.4 Types of outcome measurements

Primary outcome is HLCBC proliferation, as measured by MTT assay kit.

Secondary outcomes are HLCBC-related genes expression, including E2F transcription factor 1 and c-myc genes, as measured by Real-time polymerase chain reaction; and HLCBC-related proteins expression, consisting of c-myc mitogen-activated protein kinase 1 and cyclin D1 expression, as measured by immunofluorescence or western blot test.

### 2.3 Information sources and search strategy

This study will search electronic bibliographic databases of PUBMED, EMBASE, Cochrane Library, Scopus, Cumulative Index to Nursing and Allied Health Literature, China Biology Medicine, and China National Knowledge Infrastructure from their inception to the February 29, 2020 without restrictions to language and publication time. We will search available related CCSs or RCSs that assessed the effects of oxymatrine on the proliferation of HLCBC. A sample of search strategy for Cochrane Library is presented (Table 1). Similar search strategies for other electronic databases will be modified and applied.

**Table 1:**
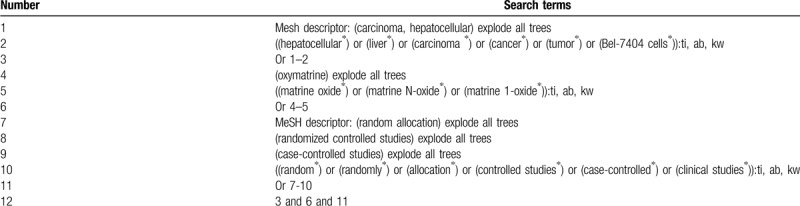
Search strategy for Cochrane Library.

In addition, we will also search related conference proceedings, and reference lists of included studies, as well as relevant reviews.

### 2.4 Study selection

Two authors will identify eligible studies from searched literatures. All titles/abstracts of potential studies will be examined and unrelated studies will be removed. The full-text of remaining studies will be cautiously read against full inclusion criteria. Any uncertainties will be solved by discussion with the help of another experienced author. All excluded studies will be recorded with reasons. The process of study selection will be shown in a Preferred Reporting Items for Systematic Reviews and Meta-Analysis flow chart.

### 2.5 Data collection and management

Two authors will independently collect data using a standard data extraction sheet. It includes study information (title, first author, year of publication, et al), types of studies (CCSs or RCSs), sample size, information of HLCBC, details of intervention and controls (dosage, frequency, et al), outcomes, and other relevant data. Any disagreements will be resolved by consultation with another experienced author. If any missing or unclear data will be identified, we will contact primary authors to request them.

### 2.6 Methodological quality assessment

Two authors will independently evaluate the methodological quality for each included study. Any divergences will be settled by another author through discussion. The methodological quality for CCSs will be appraised by Newcastle-Ottawa Scale, and that for RCSs will be examined by Cochrane risk of bias tool.

### 2.7 Statistical analysis

We will conduct statistical analyses using RevMan 5.3 software. The treatment effects of dichotomous data will be expressed as risk ratio and 95% confidence intervals, and those of continuous data will be presented as mean difference or standardized mean difference and 95% confidence intervals. *I*^2^ test will be utilized to identify heterogeneity across eligible studies. *I*^2^ ≤ 50% suggests homogeneity, and we will place a fixed-effects model to pool data. We will carry out a meta-analysis when necessary. *I*^2^ > 50% means obvious heterogeneity, and we will employ a random-effects model to synthesize data. Additionally, a subgroup analysis will be investigated to explore sources of considerable heterogeneity.

### 2.8 Additional analysis

#### 2.8.1 Subgroup analysis

A subgroup analysis will be conducted according to the different types of studies, study characteristics, and types of intervention and comparators.

#### 2.8.2 Sensitivity analysis

A sensitivity analysis will be performed to examine the robustness of study findings by eliminating low methodological quality studies.

#### 2.8.3 Reporting bias

A funnel plot and Egger regression test will be carried out to check reporting bias when over 10 studies are included.

### 2.9 Dissemination and ethics

This study will not analyze any individual patient data, thus, no ethical approval is needed. We will publish this study on a peer-reviewed journal or conference presentation.

## 3 Discussion

Previous studies reported that oxymatrine had an effect on the proliferation of HLCBC.^[16–19]^ However, there is still no convinced evidence of oxymatrine on the proliferation of HLCBC at evidence-based medicine level. Thus, this study will examine whether oxymatrine is effective on the proliferation of HLCBC. It will search present literature sources to systematically investigate the effects of oxymatrine on proliferation of HLCBC. The results of this study may provide helpful evidence to fulfill research gaps and opportunities for future research.

## Author contributions

**Conceptualization:** Zhi-ye Liu, Qing Xue, Xiu-sheng Qu.

**Data curation:** Jing Li, Hai-bo Yu, Qing Xue, Xiu-sheng Qu.

**Formal analysis:** Zhi-ye Liu, Hai-bo Yu, Qing Xue.

**Funding acquisition:** Xiu-sheng Qu.

**Investigation:** Xiu-sheng Qu.

**Methodology:** Zhi-ye Liu, Hai-bo Yu, Qing Xue.

**Project administration:** Xiu-sheng Qu.

**Resources:** Jing Li, Zhi-ye Liu, Hai-bo Yu, Qing Xue.

**Software:** Jing Li, Zhi-ye Liu, Hai-bo Yu, Qing Xue.

**Supervision:** Jing Li, Xiu-sheng Qu.

**Validation:** Zhi-ye Liu, Hai-bo Yu, Xiu-sheng Qu.

**Visualization:** Jing Li, Zhi-ye Liu, Hai-bo Yu, Qing Xue, Xiu-sheng Qu.

**Writing – original draft:** Jing Li, Zhi-ye Liu, Qing Xue, Xiu-sheng Qu.

**Writing – review & editing:** Jing Li, Zhi-ye Liu, Hai-bo Yu, Xiu-sheng Qu.
